# Portable Microelectrochemical Sensors for Rapid and Sensitive Determination of Hesperidin in *Citrus reticulate* ‘Chachi’ Peel

**DOI:** 10.3390/molecules28145316

**Published:** 2023-07-10

**Authors:** Hong-Qi Xia, Wanbing Chen, Diyang Qiu, Jiwu Zeng

**Affiliations:** Key Laboratory of South Subtropical Fruit Biology and Genetic Resource Utilization (MARA), Guangdong Province Key Laboratory of Tropical and Subtropical Fruit Tree Research, Institute of Fruit Tree Research, Guangdong Academy of Agricultural Sciences, Guangzhou 510640, China

**Keywords:** microelectrochemical sensor, hesperidin, *Citrus reticulata* “chachi”

## Abstract

Portable and low-cost analytical devices are essential for rapid detection of bioactive substrates in agricultural products. This study presents the first highly integrated microelectrochemical sensor based on pencil graphite for rapid and sensitive detection of hesperidin in *Citrus reticulate* ‘Chachi’ peel. The surface morphology and characterization as well as the electrochemical property of pencil graphite was investigated and discussed. A high electrocatalytic efficiency of hesperidin has been found at used pencil graphite-based microelectrodes. Kinetic analysis was carried out to further understand the electrochemical process of hesperidin at a pencil graphite microelectrode. Consequently, a portable and highly-integrated microelectrochemical sensor exhibits a sensitivity of 0.7251 μA cm^−2^ μM^−1^ and a detection limit as low as 25 nM (S/N = 3), and high selectivity was fabricated. Proposed microelectrochemical sensors were applied to electrochemically determinate the hesperidin content in the extract of *Citrus reticulata* “chachi” peel. As a result, the concentration of hesperidin in the actual real sample detected electrochemically with the proposed portable and low-cost microelectrochemical sensors is highly consistent to that obtained with a common chromatographic method, thus indicating the good reliability and that it can be used in practical applications.

## 1. Introduction

Portable analytical devices for rapid and sensitive detection of bioactive compounds in agricultural product are highly desired in food processing, pharmaceutical industries, as well as in modern agricultural production. Hesperidin (hesperetin-7-O-rutinoside), one of the most important flavonoids frequently found in citrus fruit, has attracted increasing attention in the past few years owing to its health effects not only as a main ingredient in functional foods and nutraceuticals that prevent and/or ameliorate cardiovascular [1] and neurodegenerative diseases [2] but also in clinical drugs for cancer [3] and, recently, COVID-19 treatment [4]. In addition, the hesperidin content has also frequently been used as a quality index of citrus products in Chinese traditional medicine [5]. For example, according to the Pharmacopoeia of China, hesperidin is the official indicator of Citri Reticulatae Pericarpium or “Chachiensis” (known as “chenpi” in Chinese) [6], which is produced by the peel of *Citrus reticulata* “chachi” (CRC) and used not only in Chinese traditional medicine but also in culinary seasonings and tea ingredients [7]. The shortage of hesperidin might debase the nutritive quality of such an agricultural product and even seriously affect its efficacy, especially when utilized in clinical drugs. Efficient and accurate analytical methods for rapid and sensitive detection of hesperidin content in such an agricultural product, therefore, continue to be a topic of interest. Although separation-based analytical methods such as chromatography enable highly sensitive and selective hesperidin detection [8,9,10], these methods are often time-consuming and require complex procedures and costly equipment.

In contrast, electrochemical sensors, which involves interfacial electrochemical reactions at electrodes to translate the chemical information of reactants to a detectable electrical signal, benefiting from a rapid response and a simple instrument, have been widely applied in various analytes [10,11]. Hesperidin is recognized to be an antioxidant containing phenolic hydroxyl groups that could be oxidized at a suitable electrode and detected electrochemically [12]. In fact, Hu et al. first reported a voltametric method for the measurement of hesperidin by using a hanging mercury drop electrode with a detection limit of 300 nM in 1996 [13]. To avoid the use of mercury, which is environmentally unfriendly and inconvenient in practical applications, and improve the sensitivity of detection, voltametric sensors constructed using solid electrodes modified with a variety of nanomaterials have been extensively studied [14,15,16]. For example, Manasa et al. reported a nano-graphene-platelet and brilliant-green composite-modified carbon paste electrode for the quantification of hesperidin with a detection limit of 50 nM [14]. Nanomaterials often provide high sensitivity and low detection limits owing to their large specific surface area (strictly, high surface-to-weight ratio) and, more importantly, their high electrocatalytic efficiencies [15,16]. However, the preparation of nanomaterials and the fabrication of functionalized electrodes are complicated and can be expensive.

In this study, pencil graphite electrodes are recognized as attractive electrode substrates owing to their low cost, simplicity, and commercial availability, and have been used for the detection of different analytes [17]. For example, several studies have reported the use of pencil graphite electrodes (bare or treated) for the sensitive detection of bioflavonoids such as quercetin [18], diosmin [19], and hesperidin [20,21]. Particularly, David et al. reported the electrocatalysis of hesperidin at pencil graphite electrodes with different hardness (from 2B to 2H) [21] although the detailed effects of the surface morphology and the characterization of pencil graphite electrodes on the electrocatalysis of hesperidin remains unclear. Furthermore, a portable microelectrochemical sensor based on pencil graphite for rapid bioflavonoids detection has not been previously reported.

Herein, a highly integrated microelectrochemical sensor based on low-cost pencil graphite for hesperidin detection is first reported. The surface morphology and characterization of pencil graphite were investigated using scanning electron microscopy (SEM), X-ray diffraction (XRD), Raman spectroscopy, as well as electrochemical methods. The high electrocatalytic efficiency and relatively low oxidative potential of hesperidin have been found at pencil graphite-based microelectrodes. Kinetic analysis was carried out to investigate the electro-oxidation of hesperidin at pencil graphite microelectrodes. A portable and low-cost pencil graphite-based microelectrochemical sensor (PGMS) was finally fabricated for the rapid and sensitive detection of hesperidin with a relatively low detection limit. Furthermore, to demonstrate the capability of the PGMS to the actual sample, the hesperidin contents in extracts of dried CRC peel were determined electrochemically and chromatographically. The results from the actual test demonstrate that as-prepared microelectrochemical sensors exhibit high reliability. Furthermore, the easy fabrication and low cost of the proposed microelectrochemical sensors make them very suitable for practical application.

## 2. Experimental

### 2.1. Materials and Chemicals

Pencil graphite lead with diameters of 0.5 mm produced by Shanghai M&G Stationery (ASLQ3108, with hardness of HB) were purchased from a computer network store. Hesperidin was purchased from Aladdin Biochemical Co. (Shanghai, China) with a purity of 97%. All other reagents used in this study were of analytical grade unless otherwise specified and used as received without further purification.

### 2.2. Apparatus

SEM images of the pencil graphite leads were obtained using a Hitachi S-3400N microscope operating at 5.0 kV. X-ray diffractometry (XRD) was performed using an X-ray diffractometer system (EMPYREAN), and Raman shift spectra was collected with Raman spectroscopy (RENISHAW inVia).

### 2.3. Preparation of Pencil Graphite Microelectrodes and Pencil Graphite Microelectrochemical Sensors

**Scheme 1:**
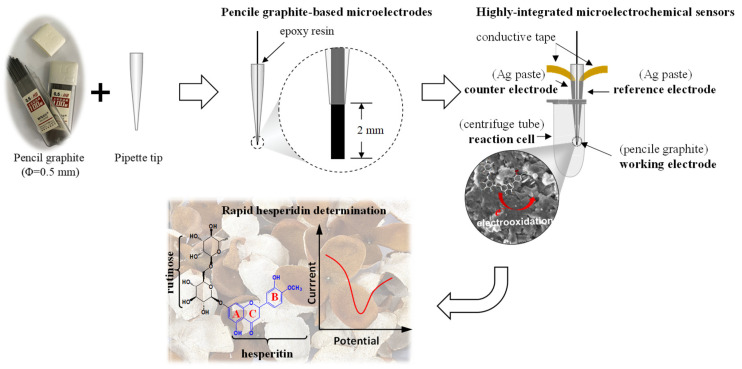
Preparation of a portable microelectrochemical sensor for rapid hesperidin determination in CRC peel.

The pencil graphite leads were used as received without any further treatment for the fabrication of microelectrodes and microelectrochemical sensors (Scheme 1). A pencil graphite lead was inserted into a micropipette tip and fixed in place using epoxy resin. One end of the pencil graphite lead was lightly polished on a filter paper to maintain a length of about 2 mm. The effective surface area of PGME was, therefore, estimated to be 0.0334 cm^2^. The prepared microelectrodes were directly used as the working electrode for further electrochemical measurements, which was carried out using an electrochemical analyzer CHI440C (Shanghai Chenhua, Shanghai, China).

To prepare the PGMS, an integrated multi-electrode assembly consistent of a pencil graphite working electrode, a self-designed counter electrode, and a reference electrode, which was fabricated by applying conductive Ag paste to the outside of micropipette tips and drying the tips at 80 °C, were prepared. The integrated multi-electrode assembly was then placed and fixed into a centrifuge tube for electrochemical measurements. A pre-prepared hole on the top of centrifuge tube was prepared for the addition of electrolyte and analyte.

### 2.4. Actual Sample Preparation

Fresh CRC fruits were collected in December 2022 from Jiangmen City, Guangdong, China. The peels of CRC were separated and dried naturally. The pre-dried CRC peel was finally dried under 45 °C to a constant weight before it was ground to a fine powder by a laboratory blender. After extraction in methanol (0.1 g mL^−1^) using an ultrasonicator for 30 min, the supernatants were collected through centrifugation at 5000 rpm for 10 min and directly used for electrochemical and chromatographic detection.

## 3. Results and Discussion

### 3.1. Surface Morphology and Characterization of Pencil Graphite

**Figure 1:**
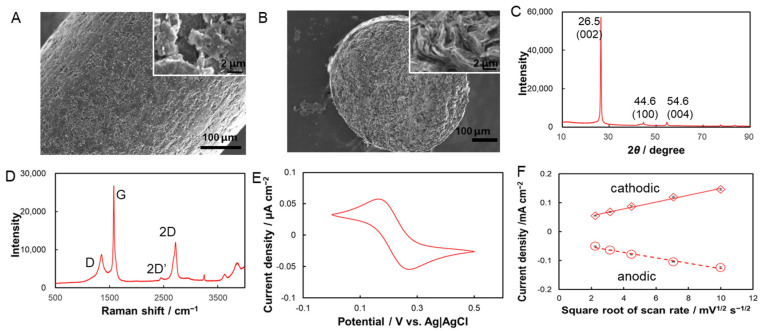
Surface morphology and analytical characterization of pencil graphite. SEM images of surface (**A**) and cross section (**B**) of pencil graphite lead with various magnifications; (**C**,**D**) shows the XRD patterns (**C**), Raman spectra (**D**) of pencil graphite leads, respectively; (**E**) cyclic voltammogram (CV) of pencil graphite electrode in 0.2 M phosphate buffer (pH 7.0) containing 1 mM K_3_Fe(CN)_6_/K_4_Fe(CN)_6_ with scan rate of 10 mV s^−1^. (**F**) Relationship between the redox peak currents detected at pencil graphite electrodes and the square root of potential scan rate.

Before electrochemical investigation, the surface morphology and microstructure of the pencil graphite electrodes were characterized using SEM. Figure 1A,B show the SEM images with various magnifications of the surfaces and a cross section of pencil graphite. It can be seen in Figure 1A that pencil graphite lead appears to have “turtleback-like” rough surfaces with crack structures. Upon magnification, large and deep gaps could be found at the surface of pencil graphite lead. However, unlike the surface, the cross-section of the pencil graphite lead displays a hierarchical layer structure with sharper graphite edges at the end (Figure 1B). The observed surface morphology of pencil graphite is consistent to that reported in the literature [22]. Such microstructure should be ascribed to the stack of graphite flakes under compress during the production of pencil leads. The rough surface and hierarchical microstructure of pencil graphite lead, with a high ratio of surface-to-volume, is very suitable to be used as electrode materials.

To further investigate the surface characterization, XRD and Raman spectra of pencil graphite were collected. Figure 1C shows that the XRD patterns of pencil graphite exhibit three main diffraction peaks at 26.5°, 44.6°, and 54.6° corresponding to the (002), (100), and (004) graphitic crystal phases, respectively. The intense diffraction peak at 26.5° implies a high degree of graphitization [23], and the faint diffraction peaks at 44.6° and 54.6° typically indicate the crystalline structure of natural graphite [24]. All peaks matched well with those of the standard pattern of pencil graphite [25]. Furthermore, the Raman spectra of pencil graphite with three prominent peaks at 1351, 1581, and 2719 cm^−1^ correspond to the D, G, 2D bands of graphite, respectively [23] (Figure 1D). The strong and significant G band at 1581 cm^−1^ represent the first-order scattering of the E2g phonon of the hexagonal sp^2^ carbon atoms, whereas the weaker *D* band at 1351 cm^−1^ is associated with the breathing mode of the *k*-point phonons of the A1g symmetry and usually indicates defects. The broad 2D band at 2719 cm^−1^, which originates from the second-order zone-boundary phonon, indicates the multilayer structures of pencil graphite. The intensity ratio of the D band to the *G* band (*I*_D_/*I*_G_), which are related to defects or disorder in the structure, is 0.28 for the used pencil graphite and is in agreement with values reported in the literature [26].

To show the electrochemical activity, cyclic voltammetry characterization of pencil graphite electrode was performed by using K_3_Fe(CN)_6_/K_4_Fe(CN)_6_ as a redox probe pair. It could be found that a pair of well-defined redox peaks with a peak-to-peak separation of 0.104 ± 0.004 V and an approximately equal peak current is clearly observed (Figure 1E). Figure S1 shows the cyclic voltammograms (CVs) of 1 mM K_3_Fe(CN)_6_/K_4_Fe(CN)_6_ with scan rates of 5–100 mV s^−1^. The well-defined redox peaks observed even at high-potential scan rates imply the efficient electron transfer at the pencil graphite electrode. Both anodic and cathodic peak currents increase linearly with the square root of the scan rate, which implies a diffusion-controlled electrode process (Figure 1F). All results indicated a quasi-reversible reaction of K_3_Fe(CN)_6_/K_4_Fe(CN)_6_ at the pencil graphite [27], which, with high electrochemical activity, thus could be used as an electrode for electrochemical measurements.

### 3.2. Electro-Oxidation of Hesperidin at a Pencil Graphite Microelectrode

**Figure 2:**
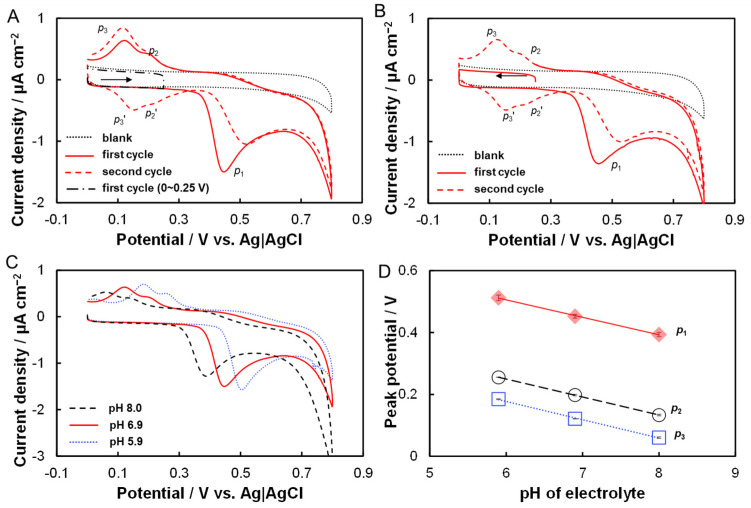
Electrocatalysis of hesperidin at PGMEs. (**A**) CVs of PGMEs in phosphate buffer (0.2 M, pH 6.9) in the absence (dotted line) and presence (solid and dashed lines) of 5 μM hesperidin; solid and dashed lines represent the first and second cycle after the addition of hesperidin; dash-dotted line shows the CVs of PGMEs in the presence of hesperidin with potential range from 0 to 0.25 V. (**B**) CVs of PGMEs in phosphate buffer (0.2 M, pH 6.9) in the absence (dotted line) and presence (solid and dashed lines) of 5 μM hesperidin with potential scan start from 0.25 V and with a negative direction. Arrows in A and B show the direction of potential scan. (**C**) CVs of PGMEs in phosphate buffer (0.2 M) containing 5 μM hesperidin with varied pH from 8.0 to 5.9. (**D**) Dependence of the peak potentials of *p*_1_, *p*_2_, and *p*_3_, on the electrolyte pH.

The electrocataysis of hesperidin at a pencil graphite microelectrode (PGME) was investigated with CVs in 0.2 M phosphate buffer solutions (PBS, pH 6.9 ± 0.1) in the absence and presence of 5 μM hesperidin (Figure 2A). No obvious redox peak is generated at the PGME in the PBS without hesperidin over the entire potential range studied (from 0 to 0.8 V vs. Ag|AgCl). After the addition of 5 μM hesperidin, a clear oxidative peak at 0.45 V (*p*_1_) and two reductive peaks at 0.20 V (*p*_2_) and 0.12 V (*p*_3_) are detected in the first cycle. Interestingly, two new oxidative peaks, which were not found at the first scan, at 0.15 V (*p*_3′_) and 0.22 V (*p*_2′_) are observed in the followed second and third cycles. The peak currents of *p*_2_ and *p*_3_ are almost equal to those of *p*_2′_ and *p*_3′_ and indicate that reversible electrode reactions occur at the PGMEs. Furthermore, both the peak current of *p*_2_/*p*_2′_ and *p*_3_/*p*_3′_ increased with the increase of a potential scan, although the peak current of *p*_1_ decreased with the increase of a potential scan. Notably, the redox peak pairs of *p*_2_/*p*_2′_ and *p*_3_/*p*_3′_ could not be detected in the first cycle when the potential range was 0 V~0.25 V (Figure 2A) or the potential scan start from 0.25 V and with a negative direction (Figure 2B). Taken together, the redox peak pairs of *p*_2_/*p*_2′_ and *p*_3_/*p*_3′_ must be generated by the oxidative product of hesperidin, which was oxidized at 0.45 V and produced an irreversible oxidative peak of *p*_1_. The oxidative current peak of *p*_1_, therefore, could be utilized for hesperidin detection.

**Scheme 2:**
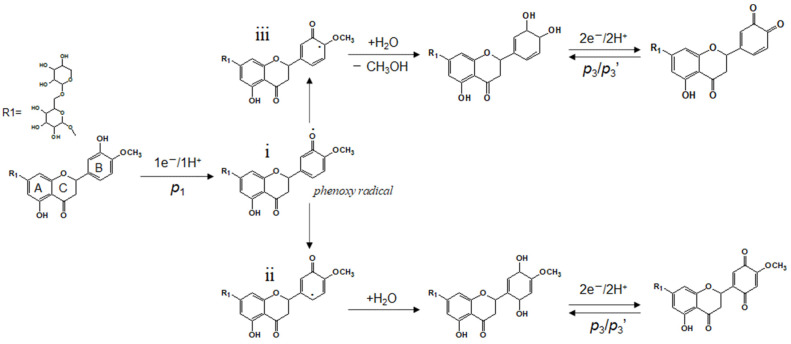
Proposed reaction process of hesperidin at pencil graphite microelectrode.

To further investigate the electrochemical reaction of hesperidin that occurred at the PGMEs, the effects of electrolyte pH on the voltammetric characterization of hesperidin were examined. It can be seen in Figure 2C that all peaks have a positive shift with the decrease of the electrolyte pH from 8.0 to 5.0, and this indicates that these electrochemical reactions involve proton transfer. Figure 2D plots the linear relationships between the peak potentials of *p*_1_, *p*_2_, *p*_3_, and electrolyte pH, respectively. As a result, the slope values for *p*_1_, *p*_2_, and *p*_3_ are −0.056 V pH^−1^ (R^2^ = 0.99), −0.058 V pH^−1^ (R^2^ = 0.99), and −0.059 V pH^−1^ (R^2^ = 0.99). All these slope values are close to the theoretical value of −0.059 V pH^−1^ according to the Nernst equation, therefore, indicating the electrode process of hesperidin at PGMEs with an equal number of electrons and protons [28]. It has been reported that the hydroxyl-group (-OH) on the flavonoids could be electrochemically oxidized and the hydroxyl-group in the B-ring is more easily oxidized than that in the A-ring [29]. Therefore, the *p*_1_ peak should be ascribed to the oxidation of the hydroxyl-group (-OH) in the B-ring of hesperidin, which involves a one-electron and one-proton process. According to the literature [30,31,32], the electrochemical oxidation of phenol lead formed a phenoxy radical (i), which is thermodynamically unstable and coexists in three isomeric forms (Scheme 2). Among them, the highest spin density of this radical is in the *ortho-* (ii)and *para*-(iii) positions, which are followed by hydrolysis to form *ortho-* and *para*- quinones [31,32]. Both *ortho*- and *para*-quinones were immediately oxidized to *ortho*- and *para*-phenol at the high electrode potential. Formed *ortho*- and *para*-phenol were then electrochemically reduced, which involves a two-electron and two-proton process and produced reductive peaks of *p*_2_ and *p*_3_ when the electrode potential shifted to a negative position and then was followed by an oxidative peak of *p*_2′_ and *p*_3′_ in the second positive scan. Furthermore, it has been reported that the redox potential of *para*-quinones is more negative than that of *ortho*-quinones [31,32]. In this viewpoint, the observed two pairs of redox peaks, *p*_3_/*p*_3′_ and *p*_2_/*p*_2′_, are generated by the electrochemical reaction of oxidized hesperidin with *para*- and *ortho*-quinones group, respectively.

It is worth mentioning here that the oxidative potential of hesperidin at the pencil graphite is 0.45 ± 0.01 V, which is significantly more negative than those of previously reported electrodes, including SnO_2_ nanoparticle/cetylpyridinium bromide-modified glassy carbon (0.56 V in pH 7.0) [33], laser-induced graphene (0.52 V in pH 6.9) [34], carbon nanotubes/nafion-modified glassy carbon (0.51 V in pH 7.0) [35], and boron-doped diamond electrodes (0.51 V in pH 7.4) [36]. The result implies that there is a high electrocatalytic efficiency of hesperidin at the PGME which, therefore, makes it suitable for sensitive hesperidin determination.

### 3.3. Portable Microelectrochemical Sensors for Hesperidin Detection

**Figure 3:**
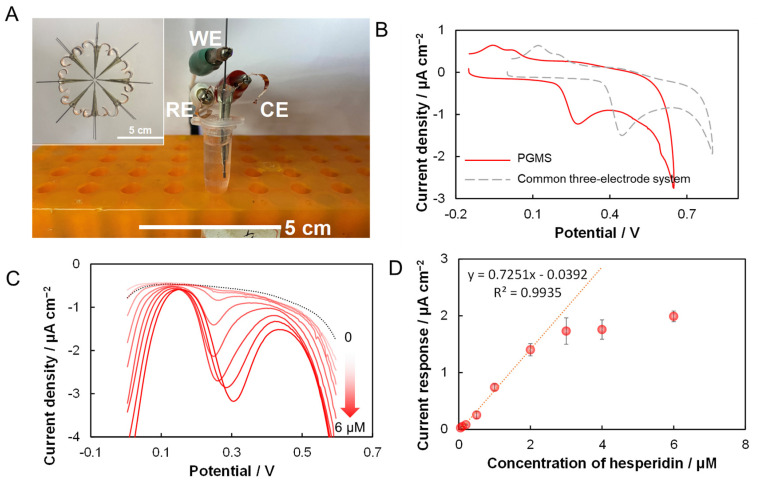
Pencil graphite-based microelectrochemical sensors (PGMSs) for hesperidin detection. (**A**) Schematic and experimental setup of PGMS for hesperidin determination; (**B**) comparison of CVs collected with PGMS and common three-electrode-system in 5 μM hesperidin contained phosphate buffer (0.2 M, pH 6.9); (**C**) DPV of PGMS with successive addition of hesperidin; (**D**) relationship between the current response and hesperidin concentration.

Owing to its high catalytic efficiency for hesperidin electro-oxidation, a portable and integrated pencil graphite-based microelectrochemical sensor (PGMS) comprising a pencil graphite micro-working electrode, and an Ag-paste counter and reference electrode, was fabricated. Figure 3A shows the experimental configuration for the electrochemical measurement in which a centrifuge tube was used as the electrolyte cell. To confirm the success of the fabrication, Figure 3B compared the CVs of 5 μM hesperidin in 0.2 M phosphate buffer (pH 6.9) collected with a typical PGMS and a common three-electrode system containing a Ag|AgCl reference electrode and a Pt wire counter electrode. It was found that both CVs had similar redox peaks, although the peak potentials at the microelectrochemical sensors were with a more negative shift than that at the common three-electrode system. The peak potential shift could be explained by the different redox potential of employed reference electrodes. Therefore, the oxidative peak observed at PGMS should be produced by the oxidation of the hesperidin.

The differential pulse voltammgrams (DPV) is a more-sensitive method than CV. The as-prepared microelectrochemical sensor was used for hesperidin determination using successive additions of certain concentrations of hesperidin standard solution into the electrolyte. DPVs with a pulse amplitude of 50 mV and a potential range of 0~0.6 V was used to determine the current response of hesperidin. As shown in Figure 3C, after the addition of hesperidin, a clear oxidative current peak was observed. The peak current increases linearly as the hesperidin concentration increases from 50 nM to 2 μM, as shown in Figure 3D. The fitted linear equation can be expressed as y = 0.7251x − 0.0392 (R^2^ = 0.9935). Therefore, the calculated sensitivity of the PGMS to hesperidin is 0.7251 μA cm^−2^ μM^−1^. The detection limit of PGMS was estimated with a signal-to-noise ratio approach (S/N = 3) to be 25 nM [37]. Table 1 compared the performances of prepared PGMS with recently reported hesperidin sensors, including nanomaterials modified electrodes, and shows that the proposed PGMS has a relatively lower detection limit than that reported in the literature [19,20,33,36,38]. The interference effects of several typical bioflavonoids, namely nobiletin and naringin, both of which are commonly found in citrus fruits, are investigated (Figure S2). As discussed in Section 3.2, the first oxidative current peak (*p*_1_) of hesperidin was proposed to be generated by the electro-oxidation of -OH group on the B-ring. Consequently, a nobiletin, an important polymethoxylated flavone found in CRC [7], without a -OH group on the B-ring thus provided a DPV without a remarkable current peak in the whole potential range from 0.1 to 0.6 V; the naringin with a *para*-OH on the B-ring displays an oxidative peak at a potential more positive than 0.4 V. The results verify the proposed electrode reaction mechanism and indicate the good selectivity of PGMSs for hesperidin detection.

### 3.4. Actual Tests in Real CRC Samples

**Figure 4:**
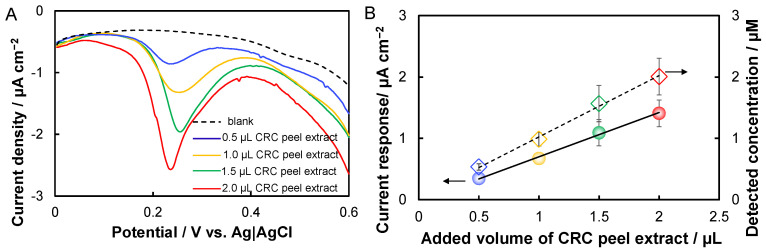
Rapid detection of hesperidin in *Citrus reticulata* ‘chachi’ (CRC) peel extract. (**A**) DPVs of the PGMSs in a phosphate buffer containing additions of different volumes of CRC peel extract; (**B**) linear relationships of the current response and the detected concentration to the added volume of CRC peel extract.

As a proof-of-concept, the capability of the prepared PGMSs for the rapid determination of hesperidin in a practical application was investigated. Figure 4A shows the DPVs of the PGMSs in a phosphate buffer using the addition of CRC extract solution with different volumes. After the addition of the CRC extract solution, the notable oxidative peak at around 0.24 V indicates the presence of hesperidin (Figure 4A). The current response increased with the volume of added CRC peel extract, indicating an increase of hesperidin. According to the fitted linear equation from Figure 3D, the final concertation of hesperidin in the electrolyte could be calculated. Figure 4B demonstrates that both the current response and the detected concentration of hesperidin increased linearly with the added volume of CRC peel extract (*R*^2^ = 0.997). It should be noted here that the original concentration of hesperidin in the prepared CRC extract solution detected with high performance liquid chromatography (HPLC) was 1.94 ± 0.03 mM. Therefore, the original concentration of hesperidin in the electrolyte with different volumes of CRC peel extract could be estimated. Table 2 summarized the experimental results in a CRC real sample. The recovery varied from 101 to 110, implying the reliability of the prepared microelectrochemical sensors.

## 4. Conclusions

In conclusion, we first fabricated a portable and low-cost microelectrochemical sensor based on pencil graphite for the rapid determination of hesperidin in CRC peel. The electro-oxidation of hesperidin occurred at a relatively low peak potential at the pencil graphite microelectrode. The electrochemical process of hesperidin at the pencil graphite microelectrodes was proposed based on kinetic analysis. A highly integrated microelectrochemical sensor was finally fabricated for the rapid detection of hesperidin that exhibits high sensitivity (0.7251 μA cm^−2^ μM^−1^), low detection limit (25 nM), as well as high selectivity. The prepared PGMSs were finally applied to detect hesperidin in real CRC samples. The concentration of hesperidin in the actual real sample detected electrochemically is highly consistent to that obtained with the common HPLC method, thus indicating the good reliability of the proposed microelectrochemical sensors and demonstrating their potential use in practical applications. Furthermore, owing to the simple fabrication process and its low cost, the potential application of the proposed pencil graphite-based microelectrochemical sensors for other analytes detection is, therefore, also highly expected.

## Figures and Tables

**Table 1:** Comparison of PGMSs with recently reported electrochemical sensors for hesperidin detection.

Electrode	LOD/μM	Linear Range/μM	Published Year
Integrated PGMS	0.025	0.05~2	This work
Electroactivated pencil graphite	0.2	0.5–10	2020 [20]
Boron-doped diamond	1.2	41–110	2020 [36]
Bare pencil graphite	0.0858	0.1~12	2021 [19]
SnO_2_ nanoparticles/cetylpyridinium bromide	0.077	0.1~10; 10–75	2021 [33]
Nanostructured porous platinum	6.6	10–400	2022 [38]

**Table 2:** Results of hesperidin detection in CRC peel extract real sample.

Original Concentration/mM (by HPLC)	Added Volume/μL	Detected Concentration/μM (by PGMS)	Recovery/%
1.94 ± 0.03	0.5	0.53 ± 0.05	110.31
	1.0	0.99 ± 0.11	101.66
	1.5	1.57 ± 0.29	107.69
	2.0	2.01 ± 0.30	103.43

## Data Availability

All relevant data have been presented in this paper.

## Supplementary Materials

The following supporting information can be downloaded at: https://www.mdpi.com/article/10.3390/molecules28145316/s1, Figure S1: CVs of 1 mM K_4_Fe(CN)_6_/K_3_Fe(CN)_6_ obtained at a typical pencil graphite microelectrode with scan rate from 5 to 100 mV s ^−1^; Figure S2: (**A**) Chemical structures of common bioflavonoids found in citrus fruits. (**B**) DPVs for various bioflavonoids in phosphate buffer.
