# Supplementary material for: Portable Microelectrochemical Sensors for Rapid and Sensitive Determination of Hesperidin in Citrus reticulate ‘Chachi’ Peel
Source: Molecules. 2023 Jul 10;28(14):5316. doi: 10.3390/molecules28145316 (PMC10384646; doi:10.3390/molecules28145316)
Supplement: Supplementary file 1 [file molecules-28-05316-s001.zip › molecules-2460723-supplementary.pdf]

Portable and low-cost microelectrochemical sensors for rapid determination of hesperidin

Hong-Qi Xia, Wanbing Chen, Diyang Qiu and Jiwu Zeng*

Key Laboratory of South Subtropical Fruit Biology and Genetic Resource Utilization (MARA), Guangdong Province Key Laboratory of Tropical and Subtropical Fruit Tree Research, Institute of Fruit Tree Research, Guangdong Academy of Agricultural Sciences, Guangzhou 510640, China

* Correspondence: Prof. Jiwu Zeng
Email-address: jiwuzeng@163.com

Supplementary Materials

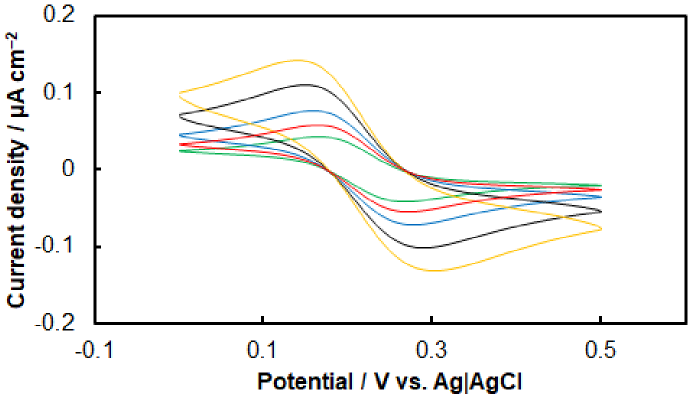

Figure S1. CVs of 1 mM $K_4Fe(CN)_6/K_3Fe(CN)_6$ obtained at a pencil graphite microelectrode with scan rate from 5 to 100 mV s^{-1}.

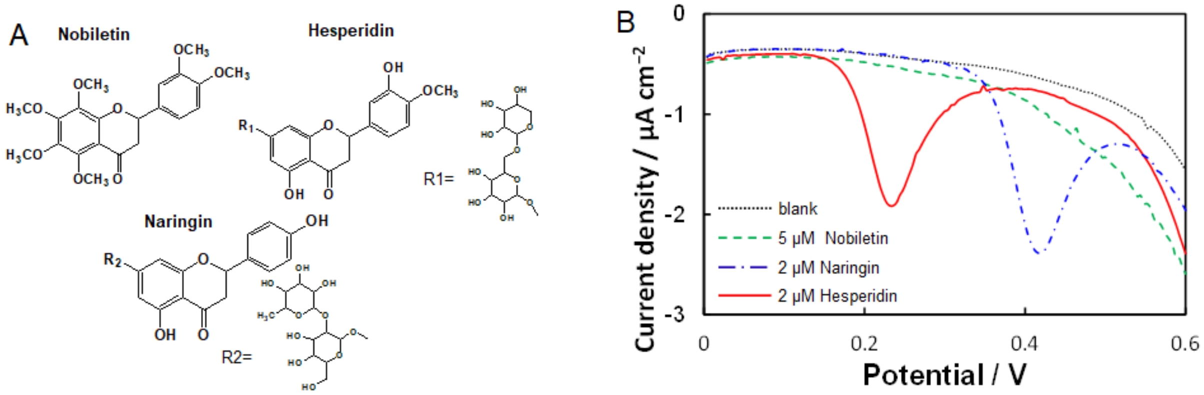

Figure S2. (**A**) Chemical structures of common bioflavonoids found in citrus fruits. (**B**) DPVs for various bioflavonoids in 0.2 M phosphate buffer (pH 6.9).
